# Application of tandem mass spectrometry in the screening and diagnosis of mucopolysaccharidoses

**DOI:** 10.1186/s13023-024-03195-w

**Published:** 2024-04-29

**Authors:** Jing-Wen Li, Shao-Jia Mao, Yun-Qi Chao, Chen-Xi Hu, Yan-Jie Qian, Yang-Li Dai, Ke Huang, Zheng Shen, Chao-Chun Zou

**Affiliations:** 1 Department of Endocrinology, the Children’s Hospital of Zhejiang University School of Medicine, Hangzhou, 310052 China; 2 Lab Center, the Children’s Hospital of Zhejiang University School of Medicine, Hangzhou, 310052 China

**Keywords:** Glycosaminoglycan, Tandem mass spectrometry, Biomarkers, Newborn screening, Mucopolysaccharidoses

## Abstract

Mucopolysaccharidoses (MPSs) are caused by a deficiency in the enzymes needed to degrade glycosaminoglycans (GAGs) in the lysosome. The storage of GAGs leads to the involvement of several systems and even to the death of the patient. In recent years, an increasing number of therapies have increased the treatment options available to patients. Early treatment is beneficial in improving the prognosis, but children with MPSs are often delayed in their diagnosis. Therefore, there is an urgent need to develop a method for early screening and diagnosis of the disease. Tandem mass spectrometry (MS/MS) is an analytical method that can detect multiple substrates or enzymes simultaneously. GAGs are reliable markers of MPSs. MS/MS can be used to screen children at an early stage of the disease, to improve prognosis by treating them before symptoms appear, to evaluate the effectiveness of treatment, and for metabolomic analysis or to find suitable biomarkers. In the future, MS/MS could be used to further identify suitable biomarkers for MPSs for early diagnosis and to detect efficacy.

## Introduction

Lysosomal storage disorder (LSD) is an inborn disorder of metabolism caused by the absence or deficiency of specific lysosomal enzymes or transporter protein activities and characterized by the accumulation of macromolecular substrates in lysosomes [7, 14, 19]. Mucopolysaccharidoses (MPSs), a subclass of LSD, are caused by a deficiency of enzymes needed for the degradation of glycosaminoglycans (GAGs) in the lysosome [40, 73, 83]. GAGs are stored in tissues and body fluids, resulting in a cumulative multitude of physical manifestations, as well as primary and secondary neurologic symptoms in patients with MPSs [27, 41, 73, 83].

MPSs can be categorized into 8 types (I, II, III, IV, VI, VII, IX, and X) based on enzyme deficiency and substrate accumulation [86]. MPS III and MPS IV can be further subdivided (Table 1) [73]. All MPSs are autosomal recessive except MPS II, which is an X-linked recessive disorder [40]. The overall incidence of MPSs varies by race and background, with an overall incidence of approximately 1 per 25,000 live births [73, 83].

**Table 1:** Types and initial symptoms of MPSs

Type	Inheritance	Gene	Deficient enzyme	Stored substrate	Initial symptoms
I	AR	*IDUA*	α-L-iduronidase	DS & HS	Skeletal abnormalities (e.g. thoracic deformities, tibial deformities, rapid or delayed growth); hernias; recurrent ear, nose, throat infections; rough facial features; hepatosplenomegaly
II	XR	*IDS*	iduronate-2-sulfatase	DS & HS	Hepatosplenomegaly; heart involvement; rough facial features; chronic respiratory tract infection; noisy and labored breathing; inguinal hernias; chronic/watery diarrhea; skeletal malformations; growth retardation; central nervous system degeneration; developmental and language delays; hearing loss and otitis media
IIIA	AR	*SGHS*	heparan N-sulfatase	HS	Restlessness, destructiveness, anxiousness, and aggressive behavior; hyperactivity and eventually severe dementia; speech delay; recurrent ear, nose, and throat infections; coarse facial features and larger protruding upper lips
B		*NAGLU*	α-N-acetylglucosaminidas		
C		*HGSNAT*	α-glucosaminidase acetyltransferase		
D		*GNS*	N-acetylglucosamine-6-sulfatase		
IVA	AR	*GALNS*	Galactosamine-6-sulfate sulfatase	KS & CS	Pectus carinatum, vertebral body deformities; gibbus deformity; pectus carinatum; short trunk dwarfism; short neck; difficult airway and chronic respiratory infections; cervical spinal cord compression; joint hypermobility; visual dysfunctions; cardiovascular involvement
B		*GLB1*	β-galactosidase	KS	
VI	AR	*ARSB*	N-acetylgalactosamine-4sulfatase	DS & CS	Coarse facial features and enlarged tongues; hepatosplenomegaly; short stature; joint stiffness; frequent upper airway infections; spinal cord compression; abnormal gait, cardiovascular disease; reduced pulmonary function
VII	AR	*GUS*	β-glucuronidase	DS、HS & CS	Hydrops fetalis; coarse facial features and large heads with scaphocephaly; progressive degeneration of development; recurrent ear infections; poor vision and hearing; sleep apnea; breathing difficulties, chronic upper respiratory tract infections; rigid chests
IX	AR	*HYAL1*	hyaluronidase-1	HA	Progressive joint manifestations and chronic inflammation and pain; short stature; flattened nose; cleft palate
X	AR	*ARSK*	arylsulfatase K	DS	Short stature; facial features and dysostosis multiplex

Mucopolysaccharidoses (MPS); autosomal recessive (AR); X-linked recessive (XR); chondroitin sulfate (CS); dermatan sulfate (DS); heparan sulfate (HS); hyaluronic acid or hyaluronan (HA); keratan sulfate (KS)

The majority of patients with MPSs are asymptomatic in the neonatal period, followed by the development of clinical signs and symptoms with involvement of multiple systems throughout the body. The symptoms and severity of MPSs vary with each patient and MPS subtype, and the average life expectancy for the majority of patients, if left untreated, ranges from 1 to 20 years [83]. Progressive clinical signs of MPSs and urinary GAGs that may be in the normal range can often lead to misdiagnosis or delayed diagnosis, resulting in poor treatment outcomes [33, 73]. Other reasons for delayed diagnosis include the rarity of the disease, inexperience of clinicians, and the lack of diagnostic equipment needed to measure lysosomal storage disease-related enzyme activity in most clinics and hospitals [19, 61, 74]. Current therapies, such as hematopoietic stem cell transplantation, enzyme replacement therapy (ERT), gene therapy, substrate reduction therapy, and pharmacologic combinations, have increased treatment options for patients with MPSs [28, 68, 73]. To optimize the efficacy of therapies such as ERT, especially for MPS patients with destructive soft tissue storage and skeletal disease with or without central nervous system involvement, it is important to initiate therapy before irreversible clinical disease develops. In general, the earlier ERT therapy is initiated in MPSs, the better the clinical outcome [21]. Early diagnosis has attracted much attention as it can lead to early treatment of patients and thus improve prognosis [63]. Some countries have included MPSs in their newborn screening programs [58].

However, there are some challenges in performing early screening and diagnosis, including finding a suitable biomarker and designing a multiplexed analytical method that can detect multiple substrates or multiple enzymes simultaneously. Multiplexed enzyme analysis by tandem mass spectrometry (MS/MS) or digital microfluidics can be performed to screen for one or more lysosomal diseases, and both platforms offer high sensitivity. Laboratory space and cost are similar for digital microfluidics and MS/MS, and the wider analytical range of MS/MS analysis provides more accurate activity values and allows for better differentiation of patients with disease-causing mutations. Another advantage of using the MS/MS method is that it is easily transferable to newborn screening laboratories [13, 66]. Here, we present MS/MS that can analyze multiple enzymes and substrates simultaneously.

## Biomarkers

Genotypes cannot be readily converted to phenotypes in many cases due to the presence of a large number of partial epigenetic DNA variants and variants of unknown pathogenic significance. Therefore, biochemical analysis remains the best first-line diagnostic method [38]. Ideal markers should be specific for one or more types of MPSs, help to differentiate between more and less severe disease phenotypes, correlate with disease progression, be responsive to therapy, and be easy to detect and quantify [36]. Biomarkers that differ significantly before the onset of clinical symptoms in children are useful in the screening and diagnosis of MPSs and can be used to assess treatment efficacy and predict disease severity [3, 40]; Kubaski, de Oliveira Poswar, et al [42]. Trim et al [85]. It is critical to identify sensitive and reliable biomarkers for individuals.

GAGs are linear, negatively charged polysaccharides that are rapidly transformed by lysosomal degradation, and their local concentrations are well regulated in healthy organisms [41, 75]. Since MPSs are primarily associated with the storage of GAGs, GAGs are natural biomarkers for these disorders, but GAGs are affected by environment, mutation type, age, anthropometric variables, renal function, and false-negative results (Kubaski, de Oliveira Poswar, et al [42]. Total GAG content provides less information, as different MPS subtypes may exhibit elevated levels of specific types of GAGs [60]. Depending on the specific enzyme defect, GAGs accumulate in the form of different isomers: dermatan sulfate (DS), heparan sulfate (HS), keratan sulfate (KS), chondroitin sulfate (CS), and hyaluronic acid (HA). This accumulation is influenced by residual levels of enzymatic activity, type of genetic variation, and environmental factors (Kubaski, de Oliveira Poswar, et al [42]. For example, the biomarker for MPS VI is DS (Lin, Lee, et al [46]. , Tebani et al [79]. Some researchers have evaluated KS levels in blood and urine for MPS other than MPS IV. Plasma KS levels were found to vary with age. Blood KS was elevated in every non-MPS IV studied (MPS I, II, III, VI, and VII). It was found through basic research that skeletal disease severity correlates with elevated levels of KS and that KS can be used as a biomarker for the severity of bone dysplasia in MPS disease [64, 82]. Potential advantages of using the GAG assay for primary testing include the fact that existing techniques such as MS/MS can cover all MPS types except MPS IX and can predict MPS types based on the elevation of specific combinations of GAGs [13]. However, there are problems with long run times, high false positive rates, and high reagent costs [13].

There are molecules that are not primary storage substances but affect cells, tissues or organs due to their primary storage. Fibroblast growth factor-2 is a molecule with a high affinity for HS, so it can be used for HS detection (Kubaski, de Oliveira Poswar, et al [42]). Heywood et al. performed a label-free quantitative proteomics study analyzing urine samples from MPS I, II, and VI and found that collagen I, fatty acid binding protein 5, nidogen-1, cartilage oligomeric matrix proteins, and insulin-like growth factor-binding protein 7 were elevated in both MPS I and II groups [36]. The serum heparin cofactor II-thrombin complex was found to be rapidly responsive to treatment in patients with MPS I, II, and VI, whereas the urinary DS/CS ratio was slower to respond, which may represent short- and long-term therapeutic outcomes, respectively [24]. Neurofibrillary light chain is a structural protein of the neuronal cytoskeleton and is a reliable biomarker of axonal injury and neurodegeneration. Neurofibrillary light chain is significantly elevated in cerebrospinal fluid (CSF) and serum of MPS II patients with neurological involvement, and they are positively correlated with CSF HS, which can be used to assess neuronal health and damage in treated MPS II patients [8]. Lysozyme B3 has been found to be a potential secondary biomarker of nervous system involvement in MPS patients [6, 72]. Elevated expression of three candidate biomarkers, matrix metalloproteinase 19, α-trypsin interinhibitor heavy chain 3, and α-1-microglobulin, was demonstrated in MPS I cartilage, and serum abundance of these molecules was found to correlate with MRI and histologic degeneration grading [92]. Although disease-specific biomarker levels can overlap with other similar diseases, the use of multiple biomarkers can help establish a diagnosis more rapidly. Examination of a range of pathophysiologically relevant markers could also help to provide insight into common downstream pathophysiologic mechanisms leading to intracellular dysfunction in these diseases [72]. Based on current findings, GAGs and their types remain the most commonly used biomarkers for MPSs.

### Screening and diagnostic processes

#### Newborn screening

**Fig. 1:**
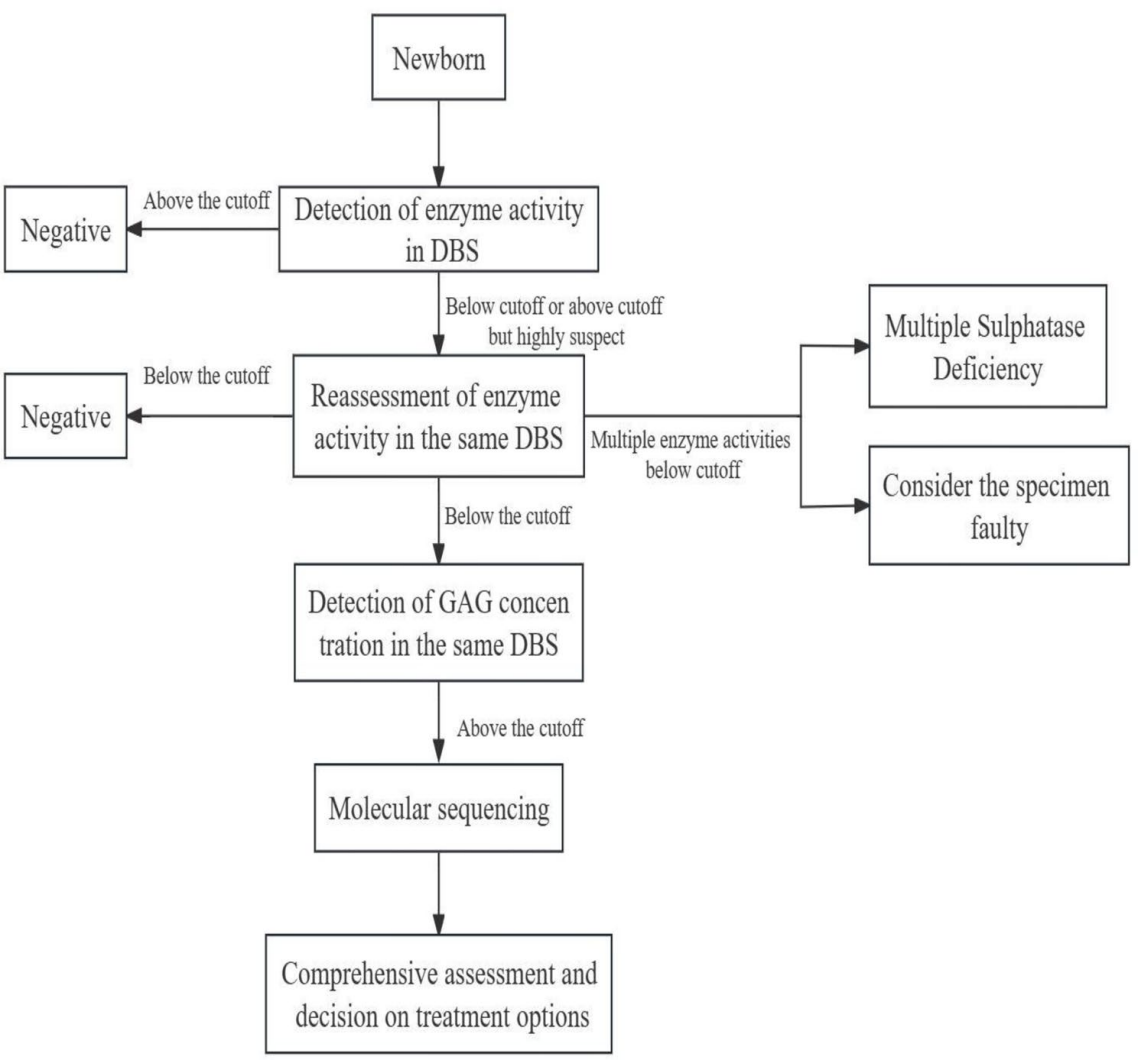
Newborn screening process

Implementation of newborn screening programs requires the combined efforts of patients and clinicians [11]. Early screening of newborns for enzymes in the dried blood spot (DBS) at birth can be used to identify high-risk patients for further definitive diagnosis [14]. In Taiwan, since the implementation of the newborn screening program, the median age at diagnosis of MPSs has decreased dramatically from 4.3 years to 0.2 years [22]. The incidence of false positives in MPS screening is high, and secondary testing of newborns can eliminate some of the false positives. GAG testing in the same DBS can serve as a secondary test [14]; Kubaski, Sousa, et al [44]). In northeastern Italy, the introduction of a secondary test for GAG on the DBS resulted in a recall of MPS I from 0.05 to 0.006% [14, 62]. GAG analysis on the DBS as a secondary analysis accurately distinguished patients with a confirmed diagnosis of MPS I from false-positive cases due to pseudodeficiency or heterozygosity, and there was no evidence of false-negative cases, but it may have missed attenuated patients [13]. There have also been pilot studies using gene sequencing as a secondary test to help identify children with pseudodeficiency and reduce referral rates [77]. However, results can take days or weeks to report, are expensive, and most newborn screening laboratories do not have the expertise to perform this secondary testing [13]. Secondary GAG testing has several important benefits over molecular testing, including lower testing costs, reduced turnaround time, and avoidance of identifying cases with uncertain molecular results [13]. There have also been pilot studies using GAG assays for primary testing. The use of GAG quantification as a primary test in newborn screening is limited by long run times (4–5 min per sample) and high reagent costs. Using gene sequencing as a primary test produces approximately half of the false positive screening results due to the presence of false defective alleles [13]. The use of postanalytical interpretation tools can also help to reduce false positives or increase specificity and positive predictive values, as shown in Fig. 1 (Kubaski, Sousa, et al [44]; Matern et al [54].

For MPS I, most programs use MS/MS as the first tier of testing to detect enzyme activity. If the enzyme activity is within the pathologic range, many laboratories will repeat the test on the same sample using the same or lower threshold. If the second result is also pathologic, a second DBS sample is collected. If the second DBS also shows α-L-iduronidase (IDUA) activity below the threshold, the neonate is referred to a clinic for confirmatory testing [1, 13, 63]. All suspected MPS II, IIIA, IVA, and VI cases must also undergo a second sulfatase test to rule out multiple sulfatase deficiency [28]. The diagnosis can be confirmed biochemically by detection of deficient enzyme activity and/or molecular genetic testing. Physical examination does not confirm the diagnosis [1, 13, 63]. Adjunctive GAG testing has several important advantages over molecular testing as an adjunctive method, including reduced testing costs, shorter turnaround times, and avoidance of identifying cases with inconclusive molecular test results that may ultimately require unnecessary clinical follow-up [13].

### Diagnosis of suspicious patients

In most countries and regions, MPSs are still not included in newborn screening, and many patients are still evaluated on the basis of clinical or family history [50, 63]. Because of the rarity and nonspecificity of MPSs, only developmental or speech delays, attention-deficit/hyperactivity disorder, or autism spectrum disorders should be screened for MPS III [81]. Recurrent rhinitis, upper airway obstruction, and coarse facial features should be considered for MPS I [32]. Other risk factors include hepato43splenomegaly, dysostosis multiplex, spondyloepiphyseal dysplasia, etc. (Kubaski, de Oliveira Poswar, et al [42]). In addition to signs and symptoms, family history is also important in suspecting MPS (Kubaski, de Oliveira Poswar, et al [42]). When considered high risk, the diagnosis can be confirmed by biochemical testing or genetic analysis (Kubaski, de Oliveira Poswar, et al [42]; Mak & Cowan [50, 81]. For example, measuring the total amount of GAGs in the urine and determining the type of GAGs in the urine can assist in diagnosis(Kubaski, de Oliveira Poswar, et al [42]; Stapleton et al [73].

Elevated total urinary GAGs can be detected by GAG electrophoresis, thin-layer chromatography, enzyme-linked immunosorbent assay, and MS/MS [67, 73]. However, there is no significant correlation between the level of mucopolysaccharides in urine and the severity of the disease, and there are some false positives and false negatives associated with qualitative and quantitative methods of determining GAGs in urine. In addition to other sulfated metabolites, normal urine contains excretory GAGs at varying and very low levels [75].

If GAGs are elevated and/or GAG species are abnormal, further blood samples should be collected to measure the activity of specific MPS enzymes to help clarify the diagnosis of the MPS type [1, 24]; Kubaski, de Oliveira Poswar, et al [42]; Saville et al [67]. Enzyme activity testing should also be performed if there is a high suspicion but the urine sample is negative for GAGs. Direct enzyme assays in peripheral blood samples may be the best way to screen for these disorders [24, 61, 81, 87]. The potential presence of enzyme pseudodeficiency limits enzyme assays, and enzyme residual levels are not associated with phenotype or disease severity [24]; Kubaski, de Oliveira Poswar, et al [42]). A combination of enzyme and GAG analysis is preferred [1]. The current recommendation is that positive results should be confirmed in leukocytes or fibroblasts. When this is not possible, enzyme analysis should be performed at least twice in DBS (in two independent samples) and/or results should be confirmed by genotyping [12].

Finally, DNA can be obtained from blood (leukocytes or dried blood spots), and specific genes can be sequenced [1]; Heon-Roberts et al [35]. Kubaski, de Oliveira Poswar, et al [42]. Saville et al [67, 84, 94]. Identification of gene mutations helps to confirm the diagnosis, identify pseudo-deficiencies, detect carrier 45status, inform genetic counseling, perform prenatal diagnosis, predict phenotypes, diagnose MPS-like syndromes, and identify patients receiving mutation-specific therapy [10, 12, 13]; Kubaski, de Oliveira Poswar, et al [42]).

**Fig. 2:**
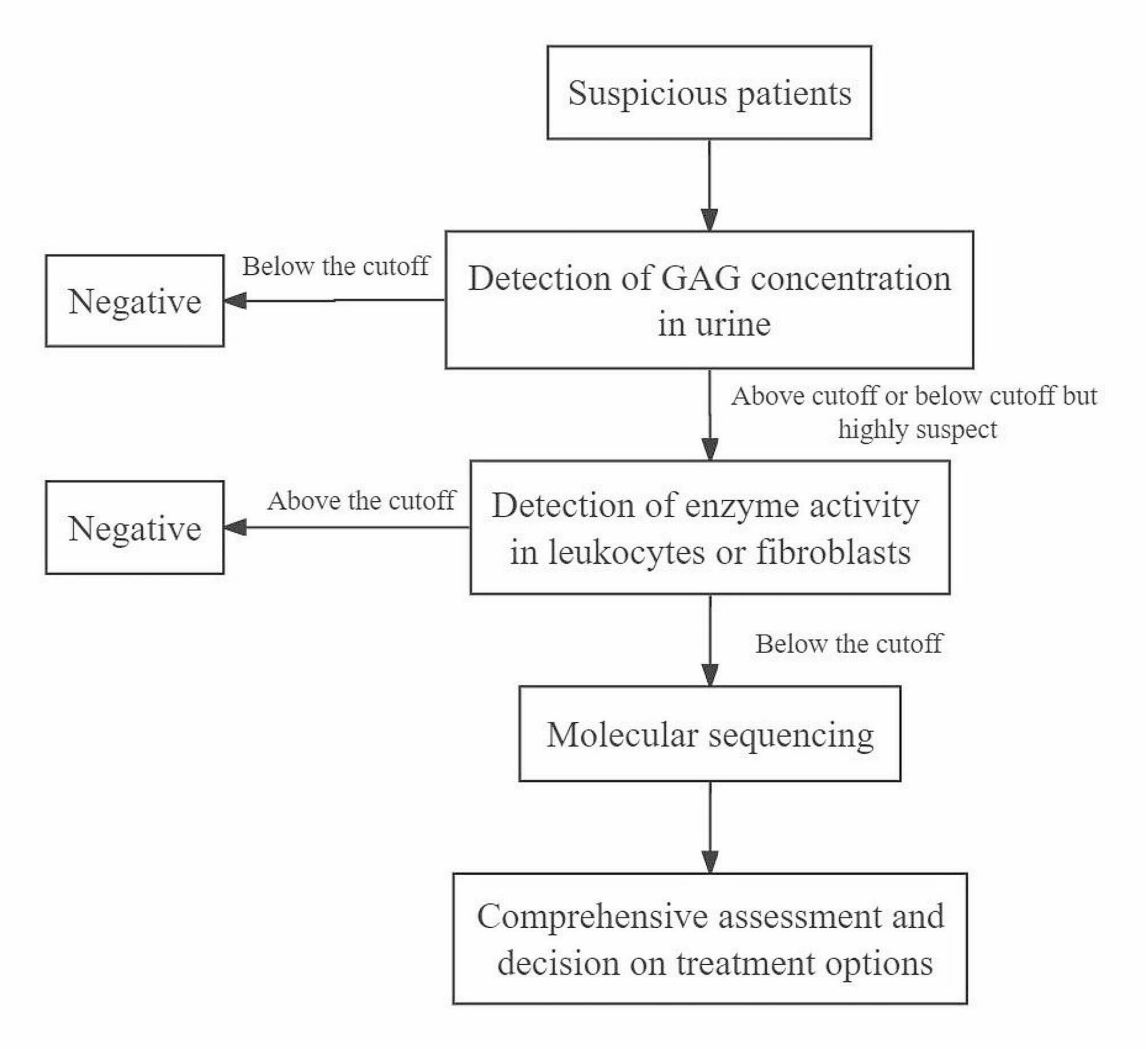
Diagnostic process for suspected patients

GAG analysis, enzyme analysis, and genetic analysis are indispensable for confirming the diagnosis of MPS patients [22]; Kubaski, de Oliveira Poswar, et al [42]. , (Fig. 2). When enzyme assays in leukocytes (or fibroblasts) are not feasible, it is recommended that diagnostic confirmation in the presence of urinary GAGs and/or enzyme assays in plasma or DBS be delayed until molecular analysis results are available. When urine is not available, measurement of GAG species in DBS by MS/MS assays is recommended to demonstrate the functional impact of enzyme deficiency and/or mutational profiling (Kubaski, de Oliveira Poswar, et al [42].

### Advantages and disadvantages of early screening and diagnosis

In the case of MPSs, early diagnosis is very important. One of them lies in the fact that early diagnosis and treatment before the appearance of irreversible pathology favors an improved prognosis [16, 55, 71]. Second, given that bone marrow transplantation is a treatment option for some patients, diagnosing MPSs early will allow clinicians to take advantage of the window of opportunity provided by the newborn’s natural immature immune system to maximize the chances of a successful transplant [55]. Third, early detection will allow parents to receive genetic counseling and the option of prenatal diagnosis in subsequent pregnancies [55]. Fourth, clinical follow-up of patients with late onset of the disease avoids misdiagnosis and unnecessary treatment, allowing children to be treated promptly at the time of their first clinical presentation [14, 16].

However, there are some problems with early screening and diagnosis, such as the diagnosis of late onset newborns, where patients and families may experience anxiety or other psychological problems after learning the results of screening [14–16]. Early screening may reveal variants of uncertain significance, which can make parental counseling very difficult and increase the risk of unnecessary medical intervention [15, 16]; Kubaski, Sousa, et al [44]). Therefore, reducing the false-positive rate is an issue that needs to be taken seriously [66]. Minter Baerg et al. [57] first reported that automated integration of covariate-adjusted reference intervals and population-based results in conjunction with secondary testing increased the false-positive rate of newborn screening for lysosomal disorders to a sustainable near-zero level. The application of postanalytical interpretation tools greatly reduces the need for secondary testing and improves the false-positive rate and positive predictive value. Existing diagnostic processes have certain problems, such as the presence of mutations of unclear significance, which make it difficult to decide on treatment options.

### Biomarker detection methods

The berry spot test is simple and inexpensive to perform but has a relatively high rate of false positives and false negatives [10]. Toluidine blue staining can only be used for qualitative analysis of GAGs [43]. Dye spectroscopy can be used to measure total GAGs in urine [40, 41, 43, 83]. Dimethyl methylene blue (DMB) is more sensitive and specific than alcian blue, and the DMB assay can be performed on dried urine filter paper samples, but it has a high rate of false negatives and cannot be used in blood samples without protease, nuclease, or hyaluronidase digestion (55). DMB cannot be used on blood samples, nor can it detect unsulfated GAGs or small GAG-derived oligosaccharides formed through GAG catabolism [2, 5, 10, 43, 47, 75]. In addition, DMB reacted to certain substances in the urine and the GAG/creatinine ratio did not stabilize until 4 days of age, suggesting that the method can be applied to day 5 neonatal urine samples [1]. False-negative results may also be related to the natural history of the disease, with some MPSs having reduced GAGs levels after growth termination [5]. Nonspectrophotometric analysis has similar sensitivity to DMB analysis but is more time consuming [10]. Thin-layer chromatography is only suitable for the initial detection of intact GAG in mixtures of limited types of samples, and its main drawbacks are the overlapping retention factors of different GAGs, which leads to misidentification of MPSs, susceptibility to age, medication, and nutritional status, among other factors, and the experience needed to correctly interpret complex patterns [30, 40, 41]. Bidirectional electrophoresis is reliable and specific, but it is more or less qualitative in nature, time consuming, and its interpretation is vague and subjective [37, 47]; Lin, Lee, et al [46]. Enzyme-linked immunosorbent assay is a qualitative and quantitative method for the determination of intact specific GAGs, but it cannot be used for the simultaneous identification of multiple GAGs [41, 43]. A variety of quantitative immunoassays, including IDUA and general markers of lysosomal dysfunction such as lysosome-associated membrane protein 1 and saponin C, have acceptable sensitivity and specificity, but these antibodies are not commercially available and the specificity and purity of the antibodies may interfere with antigen binding and antibody conjugation. More importantly, false-negative results can occur in patients with normal levels of inactive enzyme proteins; therefore, enzyme activity is more reliable as a screening indicator than enzyme protein levels [13]. A limitation of measuring enzyme activity using fluorescent artificial substrates is that each assay uses 4-methylumbelliferone as an indicator of enzyme activity and cannot be multiplied [58]. Molecular analysis is also unsuitable for high-throughput screening due to mutational heterogeneity and lack of knowledge of genotype-phenotype correlation, and in addition, rapid DNA sequencing technology is currently impractical as a primary test for newborn screening due to cost and current performance levels [13].

The magnitude of elevated blood HS and KS in patients with MPS correlates with clinical severity [40, 65, 83]. Measurements of KS and HS in blood and urine may provide useful biomarkers for early assessment of clinical severity and to monitor the efficacy of treatment for many forms of MPS [83]. Notably, GAG levels in patients with MPS decrease with age, with a decreasing gap between them and the healthy population, and testing should be performed at an early stage [37, 81, 83]. Proteomic studies by LC-MS/MS revealed that cartilage oligomeric matrix protein, insulin-like growth factor binding protein 7 and beta-galactosidase were able to differentiate between MPS II neural and non-neural clinical phenotypes [36]. In light of more studies exploring the relationship between biomarkers and disease, there are more researchers working to develop new technologies for biomarker detection.

Mass spectrometry (MS) is a technique for measuring compounds on the basis of mass-to-charge ratios with a high degree of specificity, accuracy, and sensitivity [43]. MS/MS detects abnormalities in the innate metabolism of more than 40 different amino acids, fatty acids, and organic acids [52, 54]. Analysis of GAGs requires chemical or enzymatic conversion of these polymers into small fragments that can be analyzed by MS/MS. The advantages of MS/MS include the ability to detect products with different mass change ratios and therefore analyze the results of different enzymatic reactions, high sensitivity, specificity, selectivity, and rapidity of assay, the ability to predict the type of MPSs and to monitor therapeutic efficacy, and the theoretical possibility of developing multiplex assays for a wide range of diseases [1, 58, 81]. Its drawbacks include the need for significant additional resources, additional trained professionals, ongoing service and maintenance costs, long chromatography times, the possibility of overlapping enzyme activities in carriers and some normal individuals, and the fact that for a given group of MPS patients, the clinical presentation may be very different but the differences in enzyme activity may not be significant enough to allow for an objective assessment of efficacy [10, 13, 81]. Some investigators have shown that harmonization helps to interpret enzyme activity to increase the comparability of results reported between laboratories [25], which facilitates the further establishment of appropriate cutoff values, thus reducing false positives and false negatives.

Liquid chromatography tandem mass spectrometry (LC‒MS/MS) has recently been developed as a sensitive, reproducible and accurate method for the detection of each specific GAG in blood, urine, cerebrospinal fluid, tissues and/or DBS. These methods involve enzymatic digestion of polysaccharides into disaccharides, which has the advantage of distinguishing between GAG isomers of equal molecular weight, as well as between the subclasses of HS and KS, thus identifying all types of MPSs [40]. However, this method is characterized by long incubation times, the need to use expensive reagents, and the fact that the enzyme may not be able to completely degrade all GAG molecules [29]. Another method, acid-catalyzed chemical treatment, has been used for the analysis of GAGs in urine and cerebrospinal fluid and has the advantages of a short processing time, low cost, and high reaction rate [29]. A limitation of this method is the inability to measure subclasses of GAGs [40]. In recent years, LC‒MS/MS analysis following acid-catalyzed butanolysis has emerged as a promising method for the determination of HS [34]. In 2015, Kumar et al. [45] designed an LC‒MS/MS with a high analytical range (the ratio of analytical reactions for enzymatic reactions divided by analytical reactions for nonenzymatic processes) using DBS for the detection of MPS II, IVA, and VI, thereby reducing the false-positive rate and improving diagnostic accuracy in population screening. The analytical range of this method is 1–2 orders of magnitude higher than the corresponding fluorescence analysis.

The high-throughput method absorbs the disaccharide onto a high-carbohydrate matrix, concentrating and diluting the sample prior to injection into the mass spectrometer. Elimination of chromatographic separation results in run times of only a few seconds [73]. Both LC‒MS/MS and high-throughput tandem mass spectrometry (HT-MS/MS) offer comparable sensitivity and accuracy in the simultaneous measurement of three GAGs (DS, HS, and KS) for prognosticating, diagnostic, monitoring, and screening purposes. The advantage of using LC‒MS/MS is that GAGs of the same molecular weight can be separated, thus improving the accuracy and specificity of the differential diagnosis. HT-MS/MS has higher throughput and can be used to screen for most inherited metabolic diseases, but the system cannot recognize molecules of the same molecular weight individually [1, 41, 83]. Stapleton et al. [73] suggested using HT-MS/MS for rapid mass screening followed by positive hits using LC‒MS/MS methods to characterize specific GAG subclasses.

### Applications of tandem mass spectrometry

MS/MS can be used to screen for LSDs in the neonatal period, to diagnose children in the early stages of the disease to facilitate the treatment of children before the onset of symptoms to improve the prognosis, to assess the efficacy of treatments, to perform metabolomics analyses or to search for suitable biomarkers and to predict disease phenotypes.

### For early screening and diagnosis of MPS

Detection of enzyme activity and GAGs levels in neonatal DBS samples by MS/MS, including two-layer screening of enzyme activity and combination of multiple GAGs, is helpful for early screening and diagnosis of MPSs [31, 59, 62, 67, 74, 91]. Simultaneous measurement of multiple enzyme activities by MS/MS has revealed that the prevalence of diseases such as MPS I is higher than the estimates of clinical diagnosis [69]. In recent years, several investigators have worked to develop screening techniques applicable to MPSs (Table 2). For example, Blanchard et al. [9, 26, 39] developed an assay for the screening of neonatal IDUA, galactosamine-6-sulfate sulfatase (GALNS), and N-acetylgalactosamine-4sulfatase (ARSB) activities using DBS as the enzyme source. Burton et al. [16, 20, 23, 49, 67, 70, 88, 89] developed techniques for newborn screening for simultaneous multiple lysosomal storage diseases. Khaledi et al. [38] reported the first multiplexed assay for 10 enzyme activities in dried blood spots and fibroblast lysates, which can be used for newborn screening and diagnosis of all MPSs except the extremely rare MPS IX.

**Table 2:** Application and progress of tandem mass spectrometry in recent 5 years

Researcher/ Year/ref	Sample	Characteristics	Result	Significance
Auray-Blais et al. [4]	Urine-GAG	Analyzed KS disaccharides and creatinine	All MPSI VA patients showed abnormal results before treatment compared to reference values	A good way to differentiate between MPS IVA patients and normal population
Khan et al. [40]	Urine/hematology -GAG	Assessed blood GAGs in MPS II, III, IVA and IVB, and urine GAGs in MPS IVA, IVB and VI by LC‒MS /MS	Helpful for diagnose MPSs, urinary KS is not a useful biomarker for monitoring the effectiveness of MPS IVA treatment	LC‒MS /MS can be used to track treatment effects
Tanaka et al. [76]	Brain/CSF -GAG	Quantified HS and DS in low volume samples by combining acid methanolysis and LC‒MS /MS	HS, but not DS, accumulated in the CNS. HS levels in CSF correlated with that in the brain	CSF HS Levels may be a useful biomarker for cerebral GAGs accumulation and drug efficacy in MPS II
Tebani et al. [78]	/	Targeted and untargeted metabolomics based on ultraperformance LC‒MS /MS	Major amino acid pathway impairments in MPS III, mainly arginine-proline metabolism and urea cycle metabolism	The first metabolomics-based study of MPS III and helps to elucidate the pathophysiology of MPS III.
Lin et al. [47]	Urine-GAG	Analyzing the urinary GAGs phenotype and levels among different types of MPSs by LC‒MS /MS	Different GAGs help predict phenotypes. GAG-graded biomarkers is more sensitive and reliable than DMB ratio	MS/MS can to predict phenotypes with high sensitivity
Tebani et al. [79]	/	Nontargeted metabolomics analysis	Several major amino acid pathways (arginine-proline, histidine and glutathione) are dysregulated in MPS VI	One of the first metabolic phenotyping studies of MPS VI; helpful for understanding the molecular pathophysiology of MPS
Chan et al. [18]	DBS-enzyme		Additional molecular analysis of patientss with low enzyme activity	MS/MS for MPS I, II and VI enzyme analysis for newborn screening
Saville et al. [67]	Urine-GAG	Measuring specific GAGs fragments of terminal residues associated with genetic defects	This method provides 100% specificity and sensitivity	This new urine test can diagnose 10 mucopolysaccharidosis subtypes in a single test and enables longitudinal biochemical monitoring after therapeutic interventions
Lin et al. (Lin, Lo, et al [48]). ,	Urine-GAG	Calculating GAG-derived disaccharide levels based on the amount (peak area) of CS	CS normalization produces more consistent values than creatinine normalization	The use of CS standardized suspicion reveals the actual status of DS, HS and KS
Makower et al. [51]	Brain/CSF -GAG	Determination of HS metabolites and HS digests after heparinase treatment	The relative reduction of HS in the brain of MPS IIIA mice after administration was similar, and this reduction was also reflected in the CSF	HS digests can be used in clinical studies to determine HS levels in CSF of MPS IIIA patients
Taylor et al. [77]	DBS-enzyme	Adoption of a secondary screening methodology and incorporation of a collaborative laboratory synthesis report	Discovery of pseudodeficient alleles and variants of unknown significance	Need for secondary testing to reduce follow-up burden
Kadali et al. [37]	Urine-GAG	Qualitatively and quantitatively analyzed GAGs, and perform specific enzyme analysis to confirm the diagnosis	The accuracy of the categorical regression tree model in the differential diagnosis of MPS was 96.3% and 98.3%, respectively. Thresholds for different GAGs to diagnose specific MPS types were established	Can be used for early decision making and disease diagnosis
Lin et al. (Lin, Lee, et al [46]). ,	Urine-GAG	Qualitative and quantitative analysis of GAGs with specific enzyme analysis and targeted gene sequencing to confirm diagnosis	No false-negative results for urinary DS, HS and KS using MS/MS-based methods	Established a platform for interprofessional collaboration based on risk criteria to allow for early confirmation of the diagnosis of MPSs
Burton et al. [17]	DBS-enzyme	MS/MS for measuring IDS activity in DBS	Discovery of 1 MPS II and 14 IDS pseudodeficient infants	Suggests MPS II could be included in Illinois newborn screening program
Menkovic et al. [56]	Urine-GAG	Evaporation of eluted urine samples from 21-day-old neonates with methanolysis reaction	Method validation showed high precision and accuracy for all analytes	A rapid and effective method for population-based neonatal urine screening using MS/MS is presented
Scott et al. [70]	DBS-enzyme	Analysis of five enzyme activities by a 5- plex method	The number of initial screen-positive samples using this method is low and manageable	Population-based newborn screening for related diseases is feasible
Chien et al. [20]	DBS-enzyme	8-fold analysis of 8 LSDs including MPS I, II, IIIB, IVA, VI	8-plex LSD screening test enables routine newborn screening for MPS IVA and other LSDs	Further validation of MS/MS for enzyme multiplex analysis
Wang et al. [90]	GAG	Development of high-throughput enzyme digestion assays	The method is highly sensitive	GAGs in CSF can be used as brain GAGs replacement biomarker, and this analysis can be used in future studies and applications to assess the feasibility of enzyme therapeutic effects in a variety of MPSs
Khaledi et al. [38]	DBS-enzyme	Incubation of enzyme-specific substrates with dried blood spots or fibroblast lysates	The test allows newborns to be screened and diagnosed for all MPSs except the extremely rare MPS-IX	First multiplexed assay for 10 enzyme activities in DBS and fibroblast lysates
Zhang et al. [93]	Bllod/urine/CSF-GAG	Methanolization of DS, HS, CS in serum/plasma, urine and cerebrospinal fluid to dimers	DS and HS in urine and CSF are more sensitive biomarkers for monitoring ERT therapy in MPS I patients compared to serum GAGs	Urine and CSF better for detecting disease progression
Chuang et al. [22]	Urine-GAG	Calculation of urinary DS, HS, and KS using the CS-standardized method rather than the traditional creatinine-standardized method	MS/MS-based analysis of GAG-derived disaccharides is feasible and reliable	Confirmation of the diagnosis of MPS requires quantification of GAG-derived disaccharides, and analysis of genetic variants can help predict outcome and guide treatment
Zhang et al. [92]	/	Synovial fluid and serum samples were collected from 12-month-old MPS I and healthy dogs and protein abundance was characterized using MS/MS	Elevated expression of matrix metalloproteinase 19, alpha-trypsin interrepressor heavy chain 3, and alpha-1-microglobulin confirmed in MPS I cartilage	Candidate biomarkers have potential to improve patient care
Courtney et al. [23]	DBS-enzyme	Cold-induced aqueous acetonitrile phase separation was investigated to improve the combination of 6-plex and IDS extracts	This method improves the detection of IDS products without significant impairment of other analytes	Creating a stable and time-consuming 7-plex analysis methodology

tandem mass spectrometry (MS/MS); mucopolysaccharidoses (MPSs); lysosomal storage diseases (LSDs); iduronate-2-sulfatase (IDS); glycosaminoglycan (GAG); keratan sulfate (KS); cerebrospinal fluid (CSF); heparin sulfate (HS); dermatan sulfate (DS); dimethyl methylene blue (DMB); enzyme replacement therapy (ERT); chondroitin sulfate (CS); liquid chromatography tandem mass spectrometry (LC‒MS/MS); glycosaminoglycans (GAGs); mucopolysaccharidose (MPS)

Chan et al. [18] reported that more than 100,000 DBSs were continuously collected by the Taiwan Newborn Screening Program, and their enzyme activities were measured by MS/MS. MS/MS-based highly robust enzyme assays for MPS I, II, and VI allowed for high-throughput newborn screening for these lysosomal storage disorders, and optimized thresholds combined with secondary assays eliminated false-positive results to a large extent, further suggesting that MS/MS can be used to simultaneously detect multiple enzyme activities in newborn screening. In Japan, Mashima et al. [53] validated LC‒MS/MS analysis for 3 types (MPS II, VI, and IVA) and demonstrated that disease-affected populations could be distinguished from healthy populations using LC‒MS/MS -based techniques. In 2019, Burton et al. [17] found that newborn MPS II screening avoids the delayed diagnosis experienced by many families of affected children and may improve clinical outcomes.

Screening for early neonatal MPSs has also been achieved by studying neonatal urine. Although DBS can serve as a reliable substrate for newborn screening, the main advantage of urine samples is that they are primarily noninvasive sample collection, especially for children, which can be accomplished by parents at home and is easy to mail through a plain envelope [56]. In addition, previous studies have shown that the concentration of GAGs in urine samples is higher than that in other biological fluids, such as blood and plasma, making urine a better substrate for quantifying GAGs for the early detection of MPSs [56]. However, it has also been shown in the literature that GAG in urine are not suitable biomarkers for monitoring treatment effects. Serum is more reflective of oligosaccharides in the brain and liver and therefore may provide more information about disease burden than urine (Lin, Lee, et al [46]. , . Urine GAG levels are usually lower in patients with decompensated MPS I than in patients with severe MPS I, and there has been concern that GAGs analysis may miss the diagnosis of decompensated MPS I [77]. Zhang et al. [93] quantified GAGs in serum and/or plasma using MS/MS analysis, and compared serum concentrations of DS and HS with concentrations in concomitant urine and cerebrospinal fluid samples, suggesting that urine and cerebrospinal fluid samples should be compared with serum concentrations of DS and HS. Concentrations in serum were compared with those in concurrently collected urine and cerebrospinal fluid samples, suggesting that urine remains the preferred sample type for biochemical diagnosis of MPS I [93].

For example, Menkovic et al. [56] applied the MS/MS method to screen multiple urine filter paper GAGs for absolute quantification of HS, DS, and creatinine in neonates with MPSs, which may be suitable for high-risk screening and monitoring of patients undergoing treatment for MPSs. Auray-Blais et al. [5] designed and validated a reliable tandem mass spectrometry multiplexed assay for the urinary analysis of four GAGs (DS, HS, KS, and CS) for quantitative analysis. This is the first method to simultaneously analyze KS with DS, HS, and CS-associated disaccharides using methanol depolymerization to efficiently target MPS I, II, III, IVA, IVB, VI, and VII patients [5]. A study in Taiwan found that LC‒MS/MS-based analysis of GAG-derived disaccharides was feasible and reliable. In their study, the CS standardization method was used to calculate the amount of DS, HS and KS in urine instead of the traditional creatinine standardization method. The sensitivity (true positive rate), specificity (true negative rate), and positive predictive value of this method were excellent [22]. Auray-Blais et al. [4] demonstrated, using MS/MS, that the results of enzymatic digestion of KS disaccharides and creatinine in urine specimens collected on filter paper could well differentiate between patients with MPS VI A and the normal population. To expand the possibilities of screening for LSDs, Hagemeijer et al. [30] developed an MS screening platform with high-resolution accurate mass and an open-source iterative bioinformatics pipeline. The pipeline generates comprehensive biomarker profiles and allows for extensive quality control monitoring. Using this platform, it is possible to identify multiple LSDs. Kadali et al. [37] attempted to capture MS/MS and 2D electrophoretic GAG profiles and used machine learning tools to transform them into classification and regression trees for early decision making and disease diagnosis.

### Evaluating the effectiveness of treatment

In terms of tracking treatment effects, Khan et al. [40] applied MS/MS methods to analyze GAGs in serum/plasma and urine of untreated or treated MPS patients, and showed that GAGs were higher in pediatric patients than in controls and that GAGs did not return to normal values after treatment [40]. Tanaka et al. [76] constructed a fusion protein, JR-141, which may have a therapeutic effect on GAG accumulation in the brains of MPS II patients, and quantified HS and DS using an optimized methanolysis reaction coupled with LC‒MS/MS. The results indicated that CSF HS was a potent biomarker for GAG accumulation in the brain and could be a potential tool to monitor the therapeutic effect of JR-141 in MPS II patients. Wang et al. [90] developed a high-throughput enzyme digestion assay based on plates, coupled with LC‒MS/MS. Wang et al. developed a plate-based high-throughput enzyme digestion assay combined with LC‒MS/MS to simultaneously measure heparan sulfate acetate and dermatan sulfate-derived disaccharides in tissues, CSF, and individual cell populations isolated from mouse brain, and demonstrated the feasibility of such an assay for future studies and applications in assessing the therapeutic efficacy of various MPSs. Lin et al. (Lin, Lo, et al [48]), proposed an empirical method for calculating GAG-derived disaccharide levels based on CS content, which produced more consistent values than creatinine normalization, and showed that normalization of GAG-derived disaccharide (HS, DS, and KS) content to CS better differentiated MPS patients from controls and assessed the efficacy of treatment. The catabolism of HS takes place in lysosomes, coordinated by lysosomal enzymes, sequentially from the non-reducing to the reducing end of the polysaccharide. Both HS and its metabolites can be used as markers of disease, which can be used for diagnosis and subsequent monitoring of the effects of specific therapeutic interventions. While the HS metabolite method is designed to measure the relative levels of HS metabolites, the HS digest method, which is a more time-consuming, quantitative method. It is primarily used to measure HS levels after enzymatic degradation of HS in CSF samples in clinical studies that require quantification. The researchers found that the HS metabolite method and the HS digest method showed a relative reduction in the amount of HS in the brains of mice after treatment, that this reduction was also reflected in CSF, and that the results of the two methods correlated with each other, suggesting that the HS digest method can reliably monitor the level of HS in CSF and can therefore be used for efficacy assessment [51].

### Exploring pathogenic mechanisms and new biomarkers

Tebani et al. [79] used MS/MS for untargeted metabolomics analysis and showed dysregulation of several major amino acid pathways in MPS VI. A combined analysis of targeted and untargeted metabolomics data and computerized results showed that arginine-proline, histidine, and glutathione metabolism were most affected. They also studied MPS I and III with the same approach and found that arginine, proline, and glutathione metabolism were most affected in MPS I, whereas arginine-proline metabolism and urea cycle metabolic pathways were impaired in MPS III [78, 80]. Metabolomics analysis helps to better understand disease mechanisms and identify potential biomarkers. It also helps in the development of new therapeutic options [80]. Zhang et al. [92] performed unbiased proteomic analysis using MS/MS to identify biomarkers predictive of MPS I joint disease. The candidate biomarkers identified have the potential to improve patient care [92]. Further exploration of pathogenic mechanisms can be facilitated by histologic analysis, which can contribute to the search for appropriate therapeutic options [92].

### Predicting phenotypes

MS/MS can also be useful in predicting phenotypes. Lin et al. [47] measured DS, HS and KS in urine using MS/MS and concluded that HS may lead to CNS dysfunction, DS mainly leads to soft tissue storage and skeletal involvement, and KS mainly leads to skeletal dysplasia and nonskeletal soft tissue involvement.

In conclusion, given the increasing number of therapies being developed, there is an urgent need to develop a method that can be used for clinical screening of MPSs. GAGs are reliable markers of MPSs, and finding reliable assays facilitates early diagnosis and early treatment and thus improves prognosis. MS/MS is increasingly being used to detect GAGs, and in the future, MS/MS could be used to further identify suitable biomarkers for MPSs for early diagnosis and efficacy testing. In addition, clinical staff and researchers should increase their focus on the disease.

## Data Availability

Not applicable.

## Abbreviations

MPSs: mucopolysaccharidoses
GAGs: glycosaminoglycans
MS/MS: tandem mass spectrometry
LSD: lysosomal storage disorder
ERT: enzyme replacement therapy
DS: dermatan sulfate
HS: heparan sulfate
KS: keratan sulfate
CS: chondroitin sulfate
HA: hyaluronic acid
DBS: dried blood spot
GAG: glycosaminoglycan
DMB: dimethyl methylene blue
LC‒MS/MS: liquid chromatography tandem mass spectrometry
GALNS: galactosamine-6-sulfate sulfatase
ARSB: N-acetylgalactosamine-4 sulfatase
AR: autosomal recessive
XR: X-linked recessive
IDUA: α-L-iduronidase
LSDs: lysosomal storage diseases
IDS: iduronate-2-sulfatase
CSF: cerebrospinal fluid
MS: mass spectrometry

## References

[1] Arunkumar N, Langan TJ, Stapleton M, Kubaski F, Mason RW, Singh R, Kobayashi H, Yamaguchi S, Suzuki Y, Orii K, Orii T, Fukao T, Tomatsu S (2020). Newborn screening of mucopolysaccharidoses: past, present, and future. J Hum Genet.

[2] Arunkumar N, Langan TJ, Stapleton M, Kubaski F, Mason RW, Singh R, Kobayashi H, Yamaguchi S, Suzuki Y, Orii K, Orii T, Fukao T, Tomatsu S (2020). Newborn screening of mucopolysaccharidoses: past, present, and future. J Hum Genet.

[3] Arunkumar N, Vu DC, Khan S, Kobayashi H, Can N, Oguni TB, Watanabe T, Tanaka J, Yamaguchi M, Taketani S, Ago T, Ohnishi Y, Saikia H, Álvarez S, Tomatsu S (2021). Diagnosis of mucopolysaccharidoses and mucolipidosis by Assaying Multiplex Enzymes and glycosaminoglycans. Diagnostics (Basel Switzerland).

[4] Auray-Blais C, Collerette-Tremblay J, Lavoie P (2018). UPLC–MS/MS analysis of keratan sulfate from urine samples collected on filter paper for monitoring & follow-up of Morquio A patients. Bioanalysis.

[5] Auray-Blais C, Lavoie P, Tomatsu S, Valayannopoulos V, Mitchell JJ, Raiman J, Beaudoin M, Maranda B, Clarke JTR (2016). UPLC-MS/MS detection of disaccharides derived from glycosaminoglycans as biomarkers of mucopolysaccharidoses. Anal Chim Acta.

[6] Baydakova G, Ilyushkina A, Gaffke L, Pierzynowska K, Bychkov I, Ługowska A, Wegrzyn G, Tylki-Szymanska A, Zakharova E (2020). Elevated LysoGb3 concentration in the neuronopathic forms of Mucopolysaccharidoses. Diagnostics (Basel Switzerland).

[7] Benetó N, Vilageliu L, Grinberg D, Canals I (2020). Sanfilippo Syndrome: molecular basis, Disease models and therapeutic approaches. Int J Mol Sci.

[8] Bhalla A, Ravi R, Fang M, Arguello A, Davis SS, Chiu C-L, Blumenfeld JR, Nguyen HN, Earr TK, Wang J, Astarita G, Zhu Y, Fiore D, Scearce-Levie K, Diaz D, Cahan H, Troyer MD, Harris JM, Escolar ML (2020). Characterization of fluid biomarkers reveals lysosome dysfunction and neurodegeneration in Neuronopathic MPS II patients. Int J Mol Sci.

[9] Blanchard S, Sadilek M, Scott CR, Turecek F, Gelb MH (2008). Tandem mass spectrometry for the direct assay of lysosomal enzymes in dried blood spots: application to screening newborns for mucopolysaccharidosis I. Clin Chem.

[10] Bodamer OA, Giugliani R, Wood T (2014). The laboratory diagnosis of mucopolysaccharidosis III (Sanfilippo syndrome): a changing landscape. Mol Genet Metab.

[11] Bronstein MG, Pan RJ, Dant M, Lubin B (2019). Leveraging evidence-based Public Policy and Advocacy to Advance Newborn Screening in California. Pediatrics.

[12] Brusius-Facchin AC, Malaga R, Leistner-Segal D, Giugliani R (2018). Recent advances in molecular testing to improve early diagnosis in children with mucopolysaccharidoses. Expert Rev Mol Diagn.

[13] Burlina AB, Gragnaniello V (2022). Newborn screening of mucopolysaccharidosis type I. Crit Rev Clin Lab Sci.

[14] Burlina AB, Polo G, Rubert L, Gueraldi D, Cazzorla C, Duro G, Salviati L, Burlina AP (2019). Implementation of second-tier tests in newborn screening for lysosomal disorders in North Eastern Italy. Int J Neonatal Screen.

[15] Burlina AB, Polo G, Salviati L, Duro G, Zizzo C, Dardis A, Bembi B, Cazzorla C, Rubert L, Zordan R, Desnick RJ, Burlina AP (2018). Newborn screening for lysosomal storage disorders by tandem mass spectrometry in North East Italy. J Inherit Metab Dis.

[16] Burton BK, Charrow J, Hoganson GE, Waggoner D, Tinkle B, Braddock SR, Schneider M, Grange DK, Nash C, Shryock H, Barnett R, Shao R, Basheeruddin K, Dizikes G (2017). Newborn screening for Lysosomal Storage Disorders in Illinois: the initial 15-Month experience. J Pediatr.

[17] Burton BK, Hoganson GE, Fleischer J, Grange DK, Braddock SR, Hickey R, Hitchins L, Groepper D, Christensen KM, Kirby A, Moody C, Shryock H, Ashbaugh L, Shao R, Basheeruddin K (2019). Population-based newborn screening for mucopolysaccharidosis type II in Illinois: the First Year experience. J Pediatr.

[18] Chan M-J, Liao H-C, Gelb MH, Chuang C-K, Liu M-Y, Chen H-J, Kao S-M, Lin H-Y, Huang Y-H, Kumar AB, Chennamaneni NK, Pendem N, Lin S-P, Chiang C-C (2019). Taiwan National Newborn Screening Program by Tandem Mass Spectrometry for Mucopolysaccharidoses types I, II, and VI. J Pediatr.

[19] Chen X, Qiu W, Ye J, Han L, Gu X, Zhang H (2016). Demographic characteristics and distribution of lysosomal storage disorder subtypes in Eastern China. J Hum Genet.

[20] Chien Y-H, Lee N-C, Chen P-W, Yeh H-Y, Gelb MH, Chiu P-C, Chu S-Y, Lee C-H, Lee A-R, Hwu W-L (2020). Newborn screening for Morquio disease and other lysosomal storage diseases: results from the 8-plex assay for 70,000 newborns. Orphanet J Rare Dis.

[21] Chuang C-K, Lin H-Y, Wang T-J, Huang Y-H, Chan M-J, Liao H-C, Lo Y-T, Wang L-Y, Tu R-Y, Fang Y-Y, Chen T-L, Ho H-C, Chiang C-C, Lin S-P (2018). Status of newborn screening and follow up investigations for mucopolysaccharidoses I and II in Taiwan. Orphanet J Rare Dis.

[22] Chuang C-K, Tu Y-R, Lee C-L, Lo Y-T, Chang Y-H, Liu M-Y, Liu H-Y, Chen H-J, Kao S-M, Wang L-Y, Ho H-J, Lin H-Y, Lin S-P (2022). Updated confirmatory diagnosis for mucopolysaccharidoses in Taiwanese infants and the application of Gene variants. Int J Mol Sci.

[23] Courtney E, Pickens CA, Cuthbert C, Petritis K (2023). Multiplexing Iduronate-2-Sulphatase (MPS-II) into a 7-Plex lysosomal storage disorder MS/MS assay using Cold-Induced phase separation. Int J Neonatal Screen.

[24] D’Avanzo F, Rigon L, Zanetti A, Tomanin R (2020). Mucopolysaccharidosis type II: one hundred years of Research, diagnosis, and treatment. Int J Mol Sci.

[25] Dorley MC, Dizikes GJ, Pickens CA, Cuthbert C, Basheeruddin K, Gulamali-Majid F, Hetterich P, Hietala A, Kelsey A, Klug T, Lesko B, Mills M, Moloney S, Neogi P, Orsini J, Singer D, Petritis K (2023). Harmonization of Newborn Screening results for pompe disease and mucopolysaccharidosis type I. Int J Neonatal Screen.

[26] Duffey TA, Sadilek M, Scott CR, Turecek F, Gelb MH (2010). Tandem mass spectrometry for the direct assay of lysosomal enzymes in dried blood spots: application to screening newborns for mucopolysaccharidosis VI (Maroteaux-Lamy syndrome). Anal Chem.

[27] Escolar ML, Jones SA, Shapiro EG, Horovitz DDG, Lampe C, Amartino H (2017). Practical management of behavioral problems in mucopolysaccharidoses disorders. Mol Genet Metab.

[28] Fachel FNS, Frâncio L, Poletto É, Schuh RS, Teixeira HF, Giugliani R, Baldo G, Matte U (2022). Gene editing strategies to treat lysosomal disorders: the example of mucopolysaccharidoses. Adv Drug Deliv Rev.

[29] Forni G, Malvagia S, Funghini S, Scolamiero E, Mura M, Della Bona M, Villanelli F, Damiano R, la Marca G (2019). LC-MS/MS method for simultaneous quantification of heparan sulfate and dermatan sulfate in urine by butanolysis derivatization. Clin Chim Acta.

[30] Hagemeijer MC, van den Bosch JC, Bongaerts M, Jacobs EH, van den Hout JMP, Oussoren E, Ruijter GJG (2023). Analysis of urinary oligosaccharide excretion patterns by UHPLC/HRAM mass spectrometry for screening of lysosomal storage disorders. J Inherit Metab Dis.

[31] Hall PL, Sanchez R, Hagar AF, Jerris SC, Wittenauer A, Wilcox WR (2020). Two-tiered newborn screening with post-analytical tools for pompe disease and mucopolysaccharidosis type I results in performance improvement and future direction. Int J Neonatal Screen.

[32] Hampe CS, Eisengart JB, Lund TC, Orchard PJ, Swietlicka M, Wesley J, McIvor RS (2020). Mucopolysaccharidosis type I: a review of the Natural History and Molecular Pathology. Cells.

[33] Harmatz P, Shediac R (2017). Mucopolysaccharidosis VI: pathophysiology, diagnosis and treatment. Front Bioscience (Landmark Edition).

[34] He QQ, Trim PJ, Lau AA, King BM, Hopwood JJ, Hemsley KM, Snel MF, Ferro V (2019). Synthetic disaccharide standards enable quantitative analysis of stored Heparan Sulfate in MPS IIIA Murine Brain regions. ACS Chem Neurosci.

[35] Heon-Roberts R, Nguyen ALA, Pshezhetsky AV (2020). Molecular bases of Neurodegeneration and Cognitive decline, the Major Burden of Sanfilippo Disease. J Clin Med.

[36] Heywood WE, Camuzeaux S, Doykov I, Patel N, Preece R-L, Footitt E, Cleary M, Clayton P, Grunewald S, Abulhoul L, Chakrapani A, Sebire NJ, Hindmarsh P, de Koning TJ, Heales S, Burke D, Gissen P, Mills K (2015). Proteomic Discovery and Development of a multiplexed targeted MRM-LC-MS/MS assay for urine biomarkers of Extracellular Matrix disruption in Mucopolysaccharidoses I, II, and VI. Anal Chem.

[37] Kadali S, Naushad SM, Rama Devi R, Bodiga VL (2019). Biochemical, machine learning and molecular approaches for the differential diagnosis of Mucopolysaccharidoses. Mol Cell Biochem.

[38] Khaledi H, Gelb MH (2020). Tandem Mass Spectrometry enzyme assays for Multiplex Detection of 10-Mucopolysaccharidoses in dried blood spots and fibroblasts. Anal Chem.

[39] Khaliq T, Sadilek M, Scott CR, Turecek F, Gelb MH (2011). Tandem mass spectrometry for the direct assay of lysosomal enzymes in dried blood spots: application to screening newborns for mucopolysaccharidosis IVA. Clin Chem.

[40] Khan SA, Mason RW, Giugliani R, Orii K, Fukao T, Suzuki Y, Yamaguchi S, Kobayashi H, Orii T, Tomatsu S (2018). Glycosaminoglycans analysis in blood and urine of patients with mucopolysaccharidosis. Mol Genet Metab.

[41] Khan SA, Mason RW, Kobayashi H, Yamaguchi S, Tomatsu S (2020). Advances in glycosaminoglycan detection. Mol Genet Metab.

[42] Kubaski F, de Oliveira Poswar F, Michelin-Tirelli K, Burin MG, Rojas-Málaga D, Brusius-Facchin AC, Leistner-Segal S, Giugliani R (2020). Diagnosis of Mucopolysaccharidoses. Diagnostics (Basel Switzerland).

[43] Kubaski F, Osago H, Mason RW, Yamaguchi S, Kobayashi H, Tsuchiya M, Orii T, Tomatsu S (2017). Glycosaminoglycans detection methods: applications of mass spectrometry. Mol Genet Metab.

[44] Kubaski F, Sousa I, Amorim T, Pereira D, Trometer J, Souza A, Ranieri E, Polo G, Burlina A, Brusius-Facchin AC, Netto ABO, Tomatsu S, Giugliani R (2020). Neonatal screening for MPS disorders in Latin America: a Survey of Pilot initiatives. Int J Neonatal Screen.

[45] Kumar AB, Masi S, Ghomashchi F, Chennamaneni NK, Ito M, Scott CR, Turecek F, Gelb MH, Spacil Z (2015). Tandem Mass Spectrometry has a larger Analytical Range than fluorescence assays of Lysosomal Enzymes: application to newborn screening and diagnosis of Mucopolysaccharidoses types II, IVA, and VI. Clin Chem.

[46] Lin H-Y, Lee C-L, Lo Y-T, Tu R-Y, Chang Y-H, Chang C-Y, Chiu PC, Chang T-M, Tsai W-H, Niu D-M, Chuang C-K, Lin S-P (2019). An At-Risk Population Screening Program for mucopolysaccharidoses by measuring urinary glycosaminoglycans in Taiwan. Diagnostics (Basel Switzerland).

[47] Lin H-Y, Lee C-L, Lo Y-T, Wang T-J, Huang S-F, Chen T-L, Wang Y-S, Niu D-M, Chuang C-K, Lin S-P (2018). The relationships between urinary glycosaminoglycan levels and phenotypes of mucopolysaccharidoses. Mol Genet Genom Med.

[48] Lin H-Y, Lo Y-T, Wang T-J, Huang S-F, Tu R-Y, Chen T-L, Lin S-P, Chuang C-K (2019). Normalization of glycosaminoglycan-derived disaccharides detected by tandem mass spectrometry assay for the diagnosis of mucopolysaccharidosis. Sci Rep.

[49] Liu Y, Yi F, Kumar AB, Kumar Chennamaneni N, Hong X, Scott CR, Gelb MH, Turecek F (2017). Multiplex Tandem Mass Spectrometry Enzymatic Activity Assay for Newborn Screening of the mucopolysaccharidoses and Type 2 neuronal ceroid lipofuscinosis. Clin Chem.

[50] Mak J, Cowan TM (2021). Detecting lysosomal storage disorders by glycomic profiling using liquid chromatography mass spectrometry. Mol Genet Metab.

[51] Makower Å, Arnelöf E, Andersson T, Edlund P-O, Gustavsson S, Janson J, Gelius SS, Tjernberg A (2019). Robust LC-MS/MS methods for analysis of heparan sulfate levels in CSF and brain for application in studies of MPS IIIA. Bioanalysis.

[52] Marsden D, Levy H (2010). Newborn screening of lysosomal storage disorders. Clin Chem.

[53] Mashima R, Ohira M, Okuyama T, Tatsumi A (2018). Quantification of the enzyme activities of iduronate-2-sulfatase, N-acetylgalactosamine-6-sulfatase and N-acetylgalactosamine-4-sulfatase using liquid chromatography-tandem mass spectrometry. Mol Genet Metabolism Rep.

[54] Matern D, Gavrilov D, Oglesbee D, Raymond K, Rinaldo P, Tortorelli S (2015). Newborn screening for lysosomal storage disorders. Semin Perinatol.

[55] Meikle PJ, Ranieri E, Simonsen H, Rozaklis T, Ramsay SL, Whitfield PD, Fuller M, Christensen E, Skovby F, Hopwood JJ (2004). Newborn screening for lysosomal storage disorders: clinical evaluation of a two-tier strategy. Pediatrics.

[56] Menkovic I, Marchand A-S, Boutin M, Auray-Blais C (2019). Neonatal Mass urine Screening Approach for early detection of Mucopolysaccharidoses by UPLC-MS/MS. Diagnostics (Basel Switzerland).

[57] Minter Baerg MM, Stoway SD, Hart J, Mott L, Peck DS, Nett SL, Eckerman JS, Lacey JM, Turgeon CT, Gavrilov D, Oglesbee D, Raymond K, Tortorelli S, Matern D, Mørkrid L, Rinaldo P (2018). Precision newborn screening for lysosomal disorders. Genet Medicine: Official J Am Coll Med Genet.

[58] Nakamura K, Hattori K, Endo F (2011). Newborn screening for lysosomal storage disorders. Am J Med Genet C.

[59] Oguni T, Tomatsu S, Tanaka M, Orii K, Fukao T, Watanabe J, Fukuda S, Notsu Y, Vu DC, Can TBN, Nagai A, Yamaguchi S, Taketani T, Gelb MH, Kobayashi H (2020). Validation of Liquid Chromatography-Tandem Mass Spectrometry-based 5-Plex assay for Mucopolysaccharidoses. Int J Mol Sci.

[60] Pan P, Chen M, Zhang Z, Corte AD, Souza C, Giugliani R, Pan L, Qiu Y, Amaravadi L, Wu J (2018). A novel LC-MS/MS assay to quantify dermatan sulfate in cerebrospinal fluid as a biomarker for mucopolysaccharidosis II. Bioanalysis.

[61] Parini R, Broomfield A, Cleary MA, De Meirleir L, Di Rocco M, Fathalla WM, Guffon N, Lampe C, Lund AM, Scarpa M, Tylki-Szymańska A, Zeman J (2018). International working group identifies need for newborn screening for mucopolysaccharidosis type I but states that existing hurdles must be overcome. Acta Paediatr (Oslo Norway: 1992).

[62] Polo G, Gueraldi D, Giuliani A, Rubert L, Cazzorla C, Salviati L, Marzollo A, Biffi A, Burlina AP, Burlina AB (2020). The combined use of enzyme activity and metabolite assays as a strategy for newborn screening of mucopolysaccharidosis type I. Clin Chem Lab Med.

[63] Ream MA, Lam WKK, Grosse SD, Ojodu J, Jones E, Prosser LA, Rosé AM, Comeau AM, Tanksley S, Powell CM, Kemper AR (2023). Evidence and recommendation for mucopolysaccharidosis type II newborn screening in the United States. Genet Medicine: Official J Am Coll Med Genet.

[64] Rowan DJ, Tomatsu S, Grubb JH, Montaño AM, Sly WS (2013). Assessment of bone dysplasia by micro-CT and glycosaminoglycan levels in mouse models for mucopolysaccharidosis type I, IIIA, IVA, and VII. J Inherit Metab Dis.

[65] S T, Ma G, Om TI, Am P, Aa MHMSV-CTN, La FACMTEW, Ls B, Iv LRG, Gs S, Sg FMB, K., … A, N. Heparan sulfate levels in mucopolysaccharidoses and mucolipidoses. J Inherit Metab Dis. 2005;28(5). 10.1007/s10545-005-0069-y. 10.1007/s10545-005-0069-y 16151906

[66] Sanders KA, Gavrilov DK, Oglesbee D, Raymond KM, Tortorelli S, Hopwood JJ, Lorey F, Majumdar R, Kroll CA, McDonald AM, Lacey JM, Turgeon CT, Tucker JN, Tang H, Currier R, Isaya G, Rinaldo P, Matern D (2020). A comparative effectiveness study of newborn screening methods for four lysosomal Storage disorders. Int J Neonatal Screen.

[67] Saville JT, McDermott BK, Fletcher JM, Fuller M (2019). Disease and subtype specific signatures enable precise diagnosis of the mucopolysaccharidoses. Genet Medicine: Official J Am Coll Med Genet.

[68] Sawamoto K, Stapleton M, Alméciga-Díaz CJ, Espejo-Mojica AJ, Losada JC, Suarez DA, Tomatsu S (2019). Therapeutic options for mucopolysaccharidoses: current and emerging treatments. Drugs.

[69] Scott CR, Elliott S, Buroker N, Thomas LI, Keutzer J, Glass M, Gelb MH, Turecek F (2013). Identification of infants at risk for developing fabry, pompe, or mucopolysaccharidosis-I from newborn blood spots by tandem mass spectrometry. J Pediatr.

[70] Scott CR, Elliott S, Hong X, Huang J-Y, Kumar AB, Yi F, Pendem N, Chennamaneni NK, Gelb MH (2020). Newborn screening for mucopolysaccharidoses: results of a pilot study with 100 000 dried blood spots. J Pediatr.

[71] Singh R, Chopra S, Graham C, Langer M, Ng R, Ullal AJ, Pamula VK (2020). Emerging approaches for fluorescence-based newborn screening of Mucopolysaccharidoses. Diagnostics (Basel Switzerland).

[72] Spiewak J, Doykov I, Papandreou A, Hällqvist J, Mills P, Clayton PT, Gissen P, Mills K, Heywood WE (2023). New perspectives in dried blood spot biomarkers for lysosomal Storage diseases. Int J Mol Sci.

[73] Stapleton M, Arunkumar N, Kubaski F, Mason RW, Tadao O, Tomatsu S (2018). Clinical presentation and diagnosis of mucopolysaccharidoses. Mol Genet Metab.

[74] Stapleton M, Kubaski F, Mason RW, Shintaku H, Kobayashi H, Yamaguchi S, Taketani T, Suzuki Y, Orii K, Orii T, Fukao T, Tomatsu S (2020). Newborn screening for mucopolysaccharidoses: measurement of glycosaminoglycans by LC-MS/MS. Mol Genet Metabolism Rep.

[75] Sun X, Li L, Overdier KH, Ammons LA, Douglas IS, Burlew CC, Zhang F, Schmidt EP, Chi L, Linhardt RJ (2015). Analysis of total human urinary glycosaminoglycan disaccharides by Liquid Chromatography-Tandem Mass Spectrometry. Anal Chem.

[76] Tanaka N, Kida S, Kinoshita M, Morimoto H, Shibasaki T, Tachibana K, Yamamoto R (2018). Evaluation of cerebrospinal fluid heparan sulfate as a biomarker of neuropathology in a murine model of mucopolysaccharidosis type II using high-sensitivity LC/MS/MS. Mol Genet Metab.

[77] Taylor JL, Clinard K, Powell CM, Rehder C, Young SP, Bali D, Beckloff SE, Gehtland LM, Kemper AR, Lee S, Millington D, Patel HS, Shone SM, Woodell C, Zimmerman SJ, Bailey DB, Muenzer J (2019). The North Carolina experience with mucopolysaccharidosis type I newborn screening. J Pediatr.

[78] Tebani A, Abily-Donval L, Schmitz-Afonso I, Héron B, Piraud M, Ausseil J, Zerimech F, Gonzalez B, Marret S, Afonso C, Bekri S (2018). Unveiling metabolic remodeling in mucopolysaccharidosis type III through integrative metabolomics and pathway analysis. J Translational Med.

[79] Tebani A, Abily-Donval L, Schmitz-Afonso I, Piraud M, Ausseil J, Zerimech F, Pilon C, Pereira T, Marret S, Afonso C, Bekri S (2019). Analysis of mucopolysaccharidosis type VI through integrative functional metabolomics. Int J Mol Sci.

[80] Tebani A, Schmitz-Afonso I, Abily-Donval L, Héron B, Piraud M, Ausseil J, Brassier A, De Lonlay P, Zerimech F, Vaz FM, Gonzalez BJ, Marret S, Afonso C, Bekri S (2017). Urinary metabolic phenotyping of mucopolysaccharidosis type I combining untargeted and targeted strategies with data modeling. Clin Chim Acta.

[81] Tomatsu S, Fujii T, Fukushi M, Oguma T, Shimada T, Maeda M, Kida K, Shibata Y, Futatsumori H, Montaño AM, Mason RW, Yamaguchi S, Suzuki Y, Orii T (2013). Newborn screening and diagnosis of mucopolysaccharidoses. Mol Genet Metab.

[82] Tomatsu S, Okamura K, Maeda H, Taketani T, Castrillon SV, Gutierrez MA, Nishioka T, Fachel AA, Orii KO, Grubb JH, Cooper A, Thornley M, Wraith E, Barrera LA, Laybauer LS, Giugliani R, Schwartz IV, Frenking GS, Beck M, Noguchi A (2005). Keratan sulphate levels in mucopolysaccharidoses and mucolipidoses. J Inherit Metab Dis.

[83] Tomatsu S, Shimada T, Mason RW, Montaño AM, Kelly J, LaMarr WA, Kubaski F, Giugliani R, Guha A, Yasuda E, Mackenzie W, Yamaguchi S, Suzuki Y, Orii T (2014). Establishment of glycosaminoglycan assays for mucopolysaccharidoses. Metabolites.

[84] Tong F, Wang J, Xiao R, Wu B-B, Zou C-C, Wu D-W, Wang H, Zou H, Han L-S, Yang L, Zou L, Hei M-Y, Yang R-L, Yuan T-M, Wen W, Huang X-W, Gu X-F, Yang Y-L, Huang Y-L, Zhao Z-Y (2022). Application of next generation sequencing in the screening of monogenic diseases in China, 2021: a consensus among Chinese newborn screening experts. World J Pediatrics: WJP.

[85] Trim PJ, Hopwood JJ, Snel MF (2015). Butanolysis derivatization: improved sensitivity in LC-MS/MS quantitation of heparan sulfate in urine from mucopolysaccharidosis patients. Anal Chem.

[86] Verheyen S, Blatterer J, Speicher MR, Bhavani GS, Boons G-J, Ilse M-B, Andrae D, Sproß J, Vaz FM, Kircher SG, Posch-Pertl L, Baumgartner D, Lübke T, Shah H, Kaissi A, Girisha A, Plecko B (2022). Novel subtype of mucopolysaccharidosis caused by arylsulfatase K (ARSK) deficiency. J Med Genet.

[87] Verma S, Pantoom S, Petters J, Pandey AK, Hermann A, Lukas J (2021). A molecular genetics view on mucopolysaccharidosis type II. Mutat Res Reviews Mutat Res.

[88] Wang D, Eadala B, Sadilek M, Chamoles NA, Turecek F, Scott CR, Gelb MH (2005). Tandem mass spectrometric analysis of dried blood spots for screening of mucopolysaccharidosis I in newborns. Clin Chem.

[89] Wang D, Wood T, Sadilek M, Scott CR, Turecek F, Gelb MH (2007). Tandem mass spectrometry for the direct assay of enzymes in dried blood spots: application to newborn screening for mucopolysaccharidosis II (Hunter disease). Clin Chem.

[90] Wang J, Bhalla A, Ullman JC, Fang M, Ravi R, Arguello A, Thomsen E, Tsogtbaatar B, Guo JL, Skuja LL, Dugas JC, Davis SS, Poda SB, Gunasekaran K, Costanzo S, Sweeney ZK, Henry AG, Harris JM, Henne KR, Astarita G (2020). High-throughput Liquid Chromatography-Tandem Mass Spectrometry quantification of glycosaminoglycans as biomarkers of mucopolysaccharidosis II. Int J Mol Sci.

[91] Yi F, Hong X, Kumar AB, Zong C, Boons G-J, Scott CR, Turecek F, Robinson BH, Gelb MH (2018). Detection of mucopolysaccharidosis III-A (Sanfilippo Syndrome-A) in dried blood spots (DBS) by tandem mass spectrometry. Mol Genet Metab.

[92] Zhang C, Gawri R, Lau YK, Spruce LA, Fazelinia H, Jiang Z, Jo SY, Scanzello CR, Mai W, Dodge GR, Casal ML, Smith LJ (2023). Proteomics identifies novel biomarkers of synovial joint disease in a canine model of mucopolysaccharidosis I. Mol Genet Metab.

[93] Zhang H, Dickson PI, Stiles AR, Chen AH, Le SQ, McCaw P, Beasley J, Millington DS, Young SP (2020). Comparison of dermatan sulfate and heparan sulfate concentrations in serum, cerebrospinal fluid and urine in patients with mucopolysaccharidosis type I receiving intravenous and intrathecal enzyme replacement therapy. Clin Chim Acta.

[94] Zhou J, Lin J, Leung WT, Wang L (2020). A basic understanding of mucopolysaccharidosis: incidence, clinical features, diagnosis, and management. Intractable Rare Dis Res.

